# Machine learning to analyze the factors influencing myopia in students of different school periods

**DOI:** 10.3389/fpubh.2023.1169128

**Published:** 2023-06-01

**Authors:** Hao-Jie Tong, Ze-Min Huang, Yu-Lan Li, Yi-Ming Chen, Ben Tian, Ling-Ling Ding, Li-Ling Zhu

**Affiliations:** ^1^School of Public Health, Jiamusi University, Jiamusi, Heilongjiang, China; ^2^Clinical College of Anhui Medical University, Hefei, Anhui, China

**Keywords:** myopia, machine learning, influencing factor, student, children

## Abstract

**Purpose:**

We aim to develop myopia classification models based on machine learning algorithms for each schooling period, and further analyze the similarities and differences in the factors influencing myopia in each school period based on each model.

**Design:**

Retrospective cross-sectional study.

**Participants:**

We collected visual acuity, behavioral, environmental, and genetic data from 7,472 students in 21 primary and secondary schools (grades 1–12) in Jiamusi, Heilongjiang Province, using visual acuity screening and questionnaires.

**Methods:**

Machine learning algorithms were used to construct myopia classification models for students at the whole schooling period, primary school, junior high school, and senior high school period, and to rank the importance of features in each model.

**Results:**

The main influencing factors for students differ by school section, The optimal machine learning model for the whole schooling period was Random Forest (AUC = 0.752), with the top three influencing factors being age, myopic grade of the mother, and Whether myopia requires glasses. The optimal model for the primary school period was a Random Forest (AUC = 0.710), with the top three influences being the myopic grade of the mother, age, and extracurricular tutorials weekly. The Junior high school period was an Support Vector Machine (SVM; AUC = 0.672), and the top three influencing factors were gender, extracurricular tutorial subjects weekly, and whether can you do the “three ones” when reading and writing. The senior high school period was an XGboost (AUC = 0.722), and the top three influencing factors were the need for spectacles for myopia, average daily time spent outdoors, and the myopic grade of the mother.

**Conclusion:**

Factors such as genetics and eye use behavior all play an essential role in students’ myopia, but there are differences between school periods, with those in the lower levels focusing on genetics and those in the higher levels focusing on behavior, but both play an essential role in myopia.

## 1. Introduction

Myopia is the most common refractive error and a significant public health problem worldwide, with myopia predicted to reach 49.8% of the population by 2050 (1, 2). China is one of the countries with the most serious myopia problems (3). In 2020, the prevalence of myopia among Chinese students reached 52.7%, higher than that in other countries such as Russia (42.6%) and Africa (4.7%), while China was only 10%–20% over 60 years ago (1, 4, 5). Overall, regions with higher economic levels have higher prevalence, and higher prevalence in cities than in rural areas (6, 7). The severe myopia problem in children and adolescents could be a potential crisis, given China’s vast population size.

Although myopia is prevalent among Chinese students, it remains a neglected issue among both parents and children (8). This may be attributed to inadequate knowledge about eye health, the promotion of electronic devices, and the increasing pressure of education (9–11). There is no effective treatment for myopia, which makes its progression irreversible once it occurs, Moreover, young children are more susceptible to environmental influences that predispose them to myopia than adults (7). As myopia progresses, the risk of eye diseases such as glaucoma and blindness will increase greatly, with potential financial, life, and social burdens for patients (1, 12).

Myopia is considered to result from a combination of genetic and environmental. Previous studies have shown that children with myopic parents are more likely to develop myopia than children without myopic parents (11, 13). On the other hand, environmental and behavioral factors may be necessary for myopia development, including near work, light intensity, outdoor activities, sleep, and dietary habits (14, 15). Near work has been studied extensively in recent years. A meta-analysis showed that for every additional hour of near work per week, the odds of myopia increased by 2% (16). In addition, the risk of myopia is reduced by 50% with an extra 76 min/day of outdoor activity (17). According to a study in Guangzhou, an increase of 40 min of outdoor activity at school for 6-year-olds was associated with a reduction in the prevalence of myopia over the next 3 years, possibly since distance viewing behavior and sufficiently light intensity during outdoor activities act to prevent the onset and control the development of myopia (18). Although genetic and environmental factors play an essential role in the development of myopia, the association and influence of myopia is unclear. Research has shown that genetics may significantly influence refractive development in preschool children more than environmental factors and that myopia in early school-age children may be primarily related to environmental factors (19, 20).

Machine learning has been applied to myopia research in recent years. Examples include predicting axial length of eye and identifying factors influencing myopia (21–26). Myopia classification model based on machine learning algorithm can well determine the influencing factors of students’ myopia, but the current research is limited to a specific school period. The similarities and differences in the factors affecting myopia among students in each school period need to be explored (23, 24). External factors and students’ activity habits vary with age. Therefore, in this study, we use data from primary and secondary school students’ visual acuity screening, behavior, and environment, and combine various machine learning algorithms to build a myopia classification model for each school period, the risk factors of myopia were analyzed according to the model.

## 2. Materials and methods

### 2.1. Data source

This study conducted a visual acuity screening and accompanying questionnaire in 21 public primary and secondary schools in Jiamusi City, Heilongjiang Province, between April and June 2021. Based on the population inclusion criteria (including cooperation with the survey, absence of eye disease during the survey, and no history of keratoconus treatment), data were collected from 7,472 participants. A total of 37 characteristics were included in this study, including students and parents situation, Eye health awareness and eye use behavior, and dietary status (Table 1). The purpose and procedures of the study were explained in detail to the parents or legal guardians before the study was conducted, and written informed consent, approved by the Ethics Committee of Jiamusi University and following the Declaration of Helsinki, was signed prior to the study.

**Table 1:** Assignment of myopia related factors.

Subject abbreviations	Subject	Project assignment			
		0	1	2	3
Myopia	Whether myopia or not	No	Yes		
Gender	Gender	Female	Male		
Age	Age	Continuous data			
**Parental situation**					
Edu.father	Education level of father	Low	Secondary	Higher	
Edu.mother	Education level of mother	Low	Secondary	Higher	
Myop.father	Myopic grade of father	Non-myopic	Mild	Moderate	High
Myop.mother	Myopic grade of mother	Non-myopic	Mild	Moderate	High
Parents.phone	Parents using cell phones in front of their children	Never	Sometimes	Frequently	
**Eye health awareness, eye use behavior and environment**					
Awr.range	Know the range of healthy eyesight?	No	Yes		
Awr.three.ones	Do you know the “Three Ones”?	Right	Wrong		
Habit.three.ones	Can you do the “three ones” when reading and writing?	No	Can do 1	Can do 2	Can do 3
Awr.glass	Whether myopia requires glasses	No	Yes	Do not know	
Habit.exercise	Performance of eye exercise	0 times/day	1 times/day	2 times/day	≥2 times/day
Habit.homework	Daily time for reading or doing homework	<1 h	1~2 h	≥2 h	
Habit.bad	Are there any bad reading and writing habits?	No	Yes		
Habit.time	Continuous read and write time	<40 min	40~60 min	≥60 min	
Habit.distance	The distance between eyes and book while reading	<20 cm	20~30 cm	≥30 cm	
Habit.tilt	Does the body or head tilt often when writing	No	Yes		
Habit.watch	Time to watch electronic devices per day	<1 h	1~2 h	≥ 2 h	
Habit.readLying	Whether lying down to read books or electronic products?	Never	Sometimes	Frequently	
Habit.distanceTV	Distance to watch TV	<2 m	2~3 m	≥ 3 m	
Envir.table	Is the height of the table and chairs appropriate?	Well	High	Low	
Envir.blackboard	Distance from seat to the blackboard	<2 m	2~3 m	≥ 3 m	
Envir.lightClass	Is the classroom lighting bright enough?	Appropriate	Bright	Dark	
Habit.whiteboard	Use of whiteboard time per class	<15 min	15~30 min	≥30 min	
Habit.tutorial	Extracurricular tutorial subjects weekly	0	1	2	≥3
Envir.text	Textbook font size	Well	Large	Small	
Envir.lightHome	The brightness of the lights in the home	Well	Bright	Dark	
Habit.lamp	Study at home whether to use eye protection lamps?	Never	Sometimes	Frequently	Every time
Habit.sleep	Daily sleep time	<8 h	8~10 h	≥10 h	
Habit.rest	Rest your eyes	No	Overlook	Close eyes	
Habit.outdoor	Daily outdoor activity time	<1 h	1~2 h	≥2 h	
Habit.recess	Outdoor activities during recess	Never	Sometimes	Frequently	
**Dietary status**					
Diet.veg	Intake of Vegetables	<3 times/week	3~5 times/week	Every day	
Diet.fruit	Intake of Fruit	<3 times/week	3~5 times/week	Every day	
Diet.bean	Intake of dairy and bean products	<3 times/week	3~5 times/week	Every day	
Diet.meat	Intake of eggs, meat, fish or viscera	<3 times/week	3~5 times/week	Every day	
Diet.nutrition	Intake of nutritional supplements	<3 times/week	3~5 times/week	Every day	

Because large-scale cycloplegic autorefraction is difficult to perform during the COVID-19 epidemic, and students were restricted on mobility and gatherings. Therefore, this study collected visual acuity data from participants using a visual acuity screening. Myopia is considered to occur when at least one eye is nearsighted.

This study used questionnaires to collect information about the behavior and environment in which students use their eyes. Instructions on how to complete the questionnaire are given by the researcher before the questionnaire begins, while the class teacher supervises the completion and collection of the questionnaire to ensure its quality.

### 2.2. Data pre-processing

**Figure 1:**
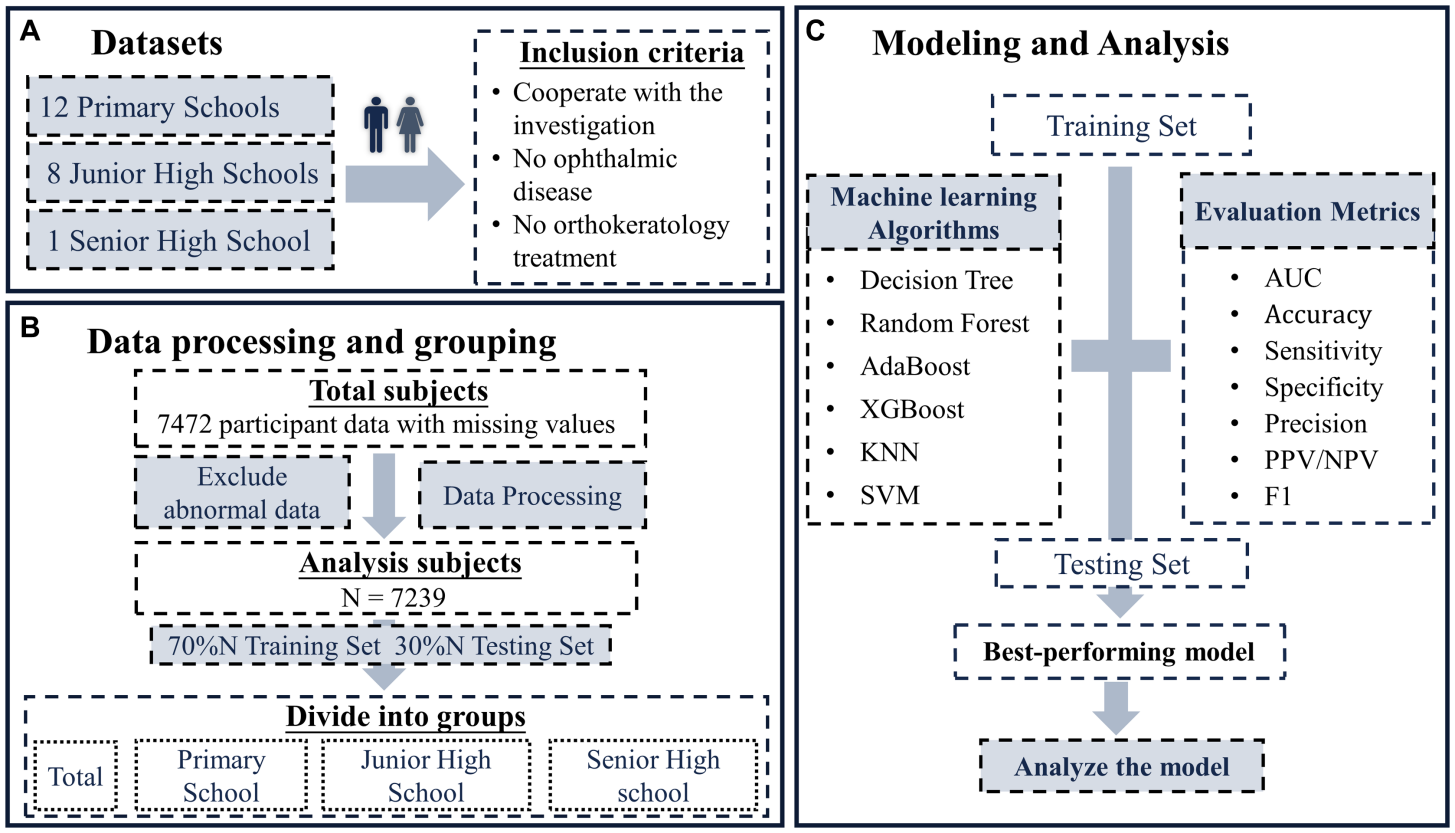
The flow chart of this study. **(A)** Study subjects, includes 12 primary schools, 8 junior high schools, and 1 senior high school; **(B)** data pre-processing and grouping, divide the data of each school period into training set and test set according to 7:3; **(C)** modeling and analysis, modeling based on multiple machine learning algorithms and analysis based on the best of these models.

The study collected data from a total of 7,472 participants, including vision screening data and questionnaire data. During data pre-processing, blank samples were removed, missing values were filled for samples with some missing values, and anomalous data were modified or deleted, resulting in a total of 7,239 samples being retained. Table 1 shows the assignment of 37 variables included in the study, identified by abbreviations due to their long names. The datasets were then divided into four datasets according to school period: the whole schooling period, primary, junior high, and senior high school period, and each dataset was further divided into a training set and a test set according to a 7:3 ratio (Figure 1). The training set is used for modeling and optimization, and the test set is used to evaluate the performance of the models so that the optimal model can be selected. Once the optimal classification model for each school period was obtained, the similarities and differences in the factors influencing myopia among students in each school period were further analyzed based on each model.

### 2.3. Development of classification model by machine learning algorithms

Machine learning is an artificial intelligence technique that improves the performance of a model by learning from experience. One of the main types is supervised learning, which refers to machine learning, where a predictive model is trained from datasets (labeled data) that have a mapping pattern from input to output. Machine learning methods are highly resistant to noisy data. They can combine features in a non-linear and highly interactive way, allowing more complex models to be built and better adapted to the characteristics of the data. A total of six standard supervised machine learning algorithms were included in this study, including Decision Trees (DT), K-Nearest Neighbor (KNN), Support Vector Machines (SVM), Random Forests (RF), eXtreme Gradient Boosting (XGboost), and Adaptive Boosting (AdaBoost).

The specific machine learning modeling process is shown in Figure 1. The training set is first used to construct a classification model based on each machine learning algorithm, and the hyperparameters are optimized to obtain each optimal model. The performance of the classification model is then evaluated using a test set based on each evaluation metric, and the optimal model is selected. Finally, the analysis was based on the optimal model for each school period, and the factors influencing myopia in each school period were analyzed, as well as the relative importance of each influencing factor.

### 2.4. Statistical and model analysis

To ensure the reliability of the machine learning models, the statistical inference was first performed on the training set, test set, and total within each school period after the dataset was split to ensure homogeneity across datasets (SPSS29.0). For the comparison of myopia rates and sex ratios between data sets, $x^{2}$ test for the comparison of multiple sample rates was used. As the age data did not follow a normal distribution, median and interquartile ranges were used to describe it, while the Kruskal-Wallis *H-*test was used to compare between groups. The machine learning modeling and analyzing steps in this study were performed using R software. Model performance was assessed using AUC, Accuracy, Sensitivity, PPV, NPV, and F1 score. For model analysis, SHAP summary plots and SHAP feature importance are used to analyze the importance of each feature in the model.

AUC is the area under the ROC curve, reflecting the accuracy of the model. Accuracy is the ratio of the number of correctly predicted samples to the total number of samples. Sensitivity is the proportion of true positive samples judged to be positive. Positive predictive value (PPV) is the proportion of samples judged to be positive that are truly positive. Negative predictive value (NPV) is the proportion of true negatives among all samples predicted to be negative. F1 score is the summed average of precision and recall. SHapley Additive explanation (SHAP) uses game theory to explain the contribution of each feature to the corresponding predicted value for each sample in the machine learning model. SHAP summary plots reflect the influence of the features in each sample. SHAP feature importance sums the absolute values of the SHAP of each identical feature in each sample and ranks them to reflect the relative importance of each feature.

## 3. Results

### 3.1. Status of datasets

Basic information about the study population is described in Table 2. A total of 7,239 students with a myopia rate of 30.709% were included in this study. The prevalence of myopia increases with age, with rates of 18.142%, 43.850%, and 48.591% among primary school, junior high school, and high school students, respectively. Division of student data from each school period into a training set (70%) and a test set (30%). The training set is used to train the model, and the test set is used to evaluate the model. Statistically inferred differences between the total, training, and test sets for each school section were not statistically significant (*p* > 0.05) and could be used for model construction and evaluation.

**Table 2:** Data division situation.

Dataset	Number of persons	Myopia rate (%)	Percentage of male (%)	Age, M (Q1~Q3), years
**The whole schooling period**				
Total	7,239	30.709	52.162	12 (10~14)
Training set	5,109	30.987	51.615	12 (10~14)
Testing set	2,130	30.051	53.474	12 (10~14)
$x^{2}$ /H		0.623	2.083	2.177
*P*		**0.732**	0.353	0.337
**Primary school period**				
Total	3,905	18.412	50.909	10 (8~11)
Training set	2,759	18.485	50.671	10 (8~11)
Testing set	1,146	18.237	51.483	10 (8~11)
$x^{2}$ /H		0.033	0.214	0.995
*P*		0.984	0.898	0.608
**Junior high school period**				
Total	2,447	43.850	51.451	14 (13~15)
Training set	1,739	43.416	52.156	14 (13~15)
Testing set	708	44.915	49.718	14 (13~15)
$x^{2}$ /H		0.459	1.198	0.004
*P*		0.795	0.549	0.998
**Senior high school period**				
Total	887	48.591	59.639	16 (16~17)
Training set	619	48.303	60.258	16 (16~17)
Testing set	268	49.254	58.209	16 (16~17)
$x^{2}$ /H		0.068	0.326	1.670
*P*		0.967	0.849	0.434

### 3.2. Performance of models

**Figure 2:**
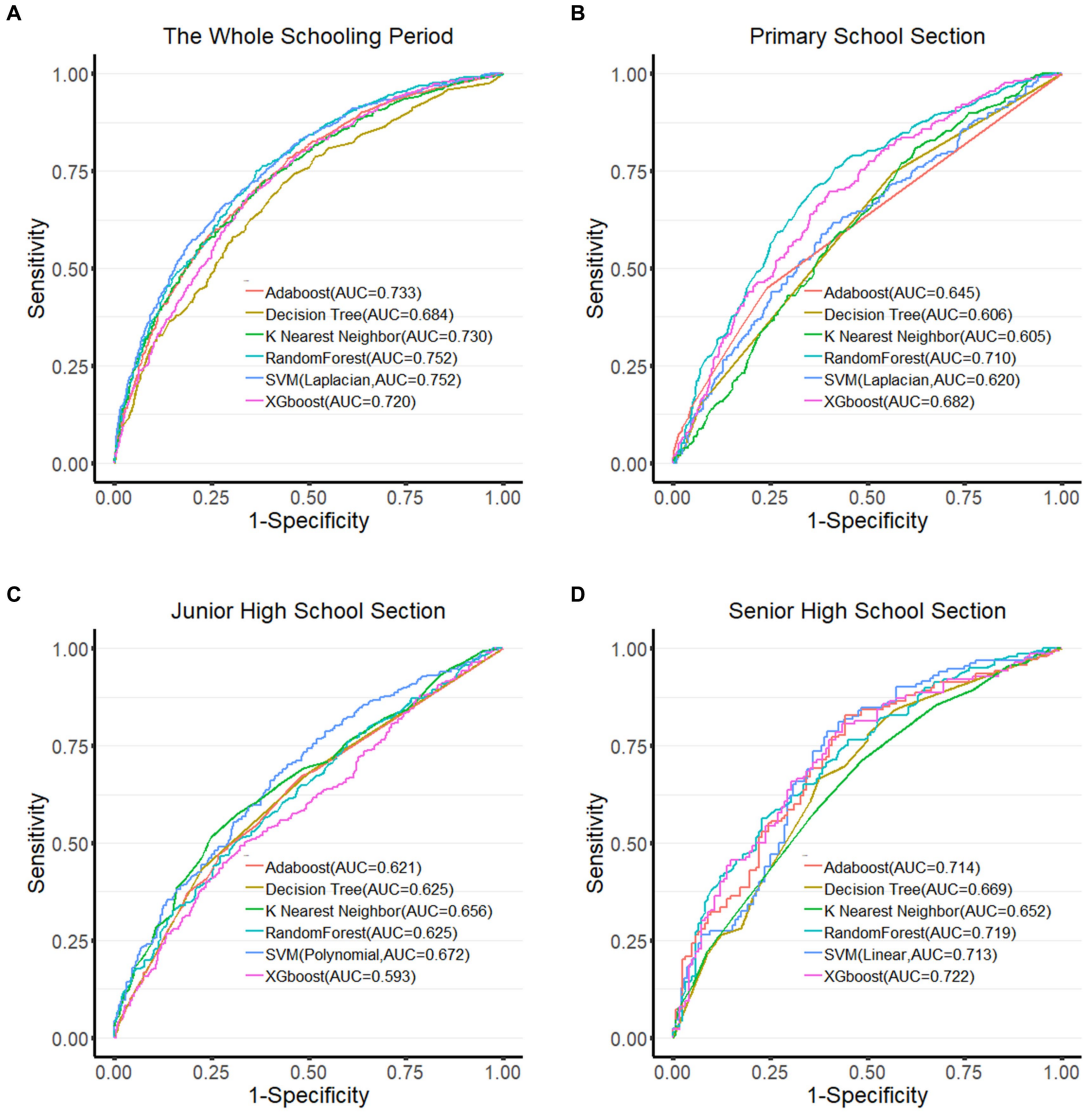
ROC curves of each algorithm in different groups. **(A)** ROC curve of the whole schooling period; **(B)** ROC curve of the primary school section; **(C)** ROC curve of the junior high school section; **(D)** ROC curve of the senior high school section. Larger the area under the ROC curve (AUC), the better the model performance.

Evaluating the model performance using the test set (Figure 2), the Random Forest algorithm performed best during the whole schooling period (AUC = 0.752) and at the primary section (AUC = 0.710). At the senior high school section (AUC = 0.722), the best performer was the XGboost algorithm. The SVM algorithm performed best in the junior high school section (AUC = 0.672). The specific results for the other evaluation indicators are presented in Table 3.

**Table 3:** Performance of the algorithm.

	Accuracy	Specificity	Sensitivity	PPV	NPV	F1
**The whole schooling period**						
Decision Tree	0.682	0.801	0.413	0.481	0.754	0.444
K Nearest Neighbor	0.730	0.953	0.230	0.683	0.735	0.344
Random Forest	0.740	0.912	0.339	0.624	0.763	0.438
SVM	0.725	0.965	0.186	0.701	0.727	0.294
AdaBoost	0.735	0.909	0.331	0.610	0.760	0.429
XGBoost	0.704	0.815	0.444	0.508	0.773	0.474
**Primary school section**						
Decision Tree	0.800	0.967	0.053	0.262	0.821	0.088
K Nearest Neighbor	0.812	0.997	0.000	0.000	0.814	0.000
Random Forest	0.809	0.989	0.018	0.227	0.816	0.035
SVM	0.815	0.000	0.000	0.000	0.815	0.000
AdaBoost	0.817	0.987	0.069	0.556	0.823	0.122
XGBoost	0.787	0.930	0.161	0.343	0.829	0.219
**Junior high school section**						
Decision Tree	0.573	0.636	0.497	0.527	0.636	0.512
K Nearest Neighbor	0.610	0.851	0.315	0.633	0.604	0.421
Random Forest	0.602	0.780	0.389	0.595	0.605	0.470
SVM	0.623	0.736	0.484	0.599	0.636	0.535
AdaBoost	0.604	0.825	0.337	0.617	0.599	0.436
XGBoost	0.591	0.699	0.462	0.561	0.609	0.507
**Senior high school section**						
Decision Tree	0.627	0.647	0.606	0.625	0.629	0.615
K Nearest Neighbor	0.608	0.640	0.576	0.608	0.608	0.592
Random Forest	0.653	0.730	0.586	0.713	0.605	0.643
SVM	0.672	0.684	0.659	0.669	0.674	0.664
AdaBoost	0.637	0.721	0.564	0.699	0.591	0.624
XGBoost	0.668	0.697	0.643	0.709	0.630	0.674

### 3.3. Analysis of risk factors for myopia in students

**Figure 3:**
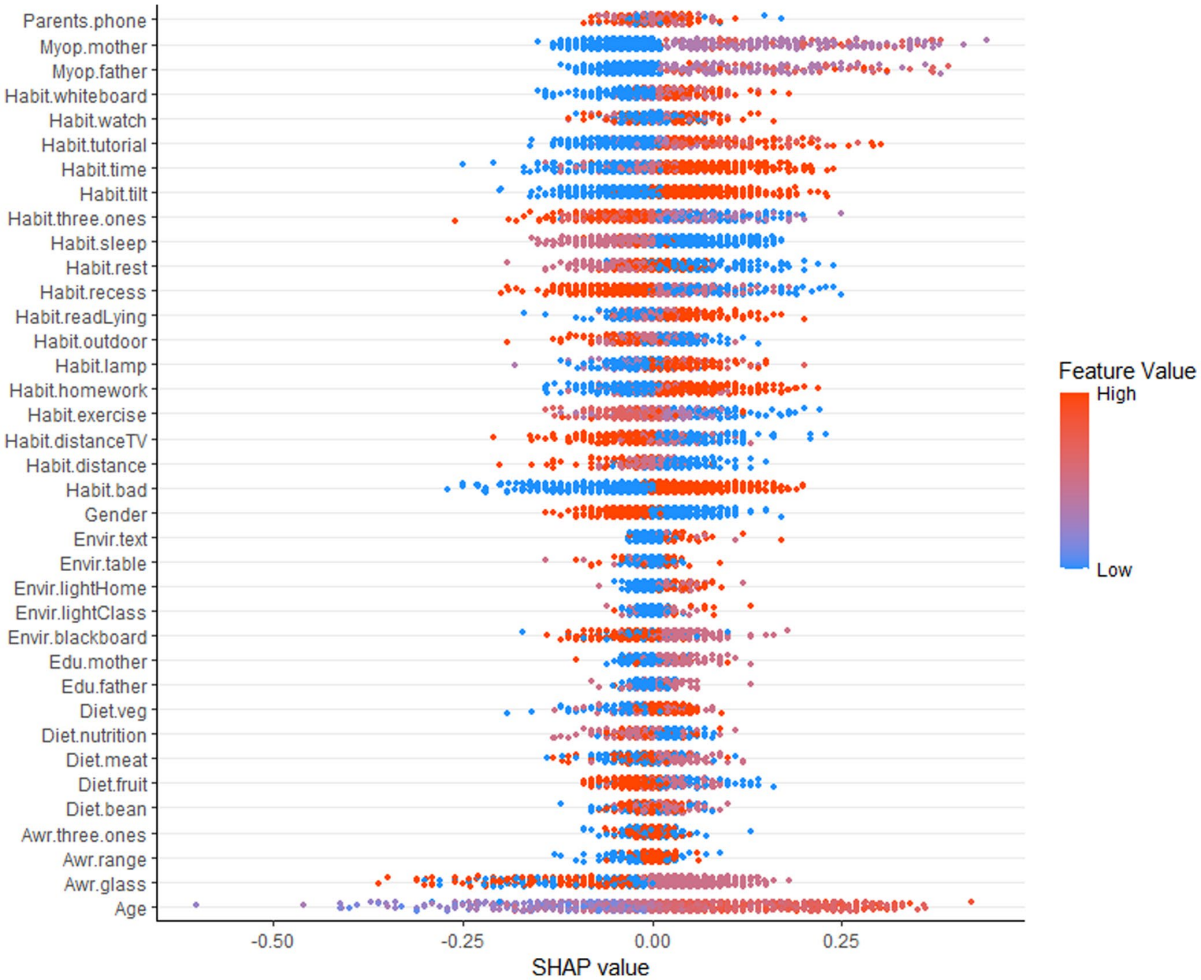
SHAP summary plot based on the whole schooling period.

Figure 3 shows a SHAP summary plot showing the contribution of each feature of the optimal myopia classification model (Random Forest algorithm) to the outcome (myopia) for the full range of students. One point per feature represents a sample, but since some points are overlapping, some data information needs to be lost. The dot’s color represents the eigenvalue’s size; the closer to red, the larger the eigenvalue. The width of the strip of dots reflects the magnitude of the effect of the feature on the result; the longer the strip, the greater the effect. The horizontal coordinate represents the SHAP value; if the SHAP value is less than 0, it has a negative effect on the result. Otherwise, it has a positive effect. As in Figure 3, on the whole schooling period, the bars for the age characteristic are the widest, reflecting the fact that age has the most significant effect on the outcome (Myopia) of all the variables and that most of the red dots are distributed to the right of the 0 value, indicating that the older the age, the greater the risk of myopia.

**Figure 4:**
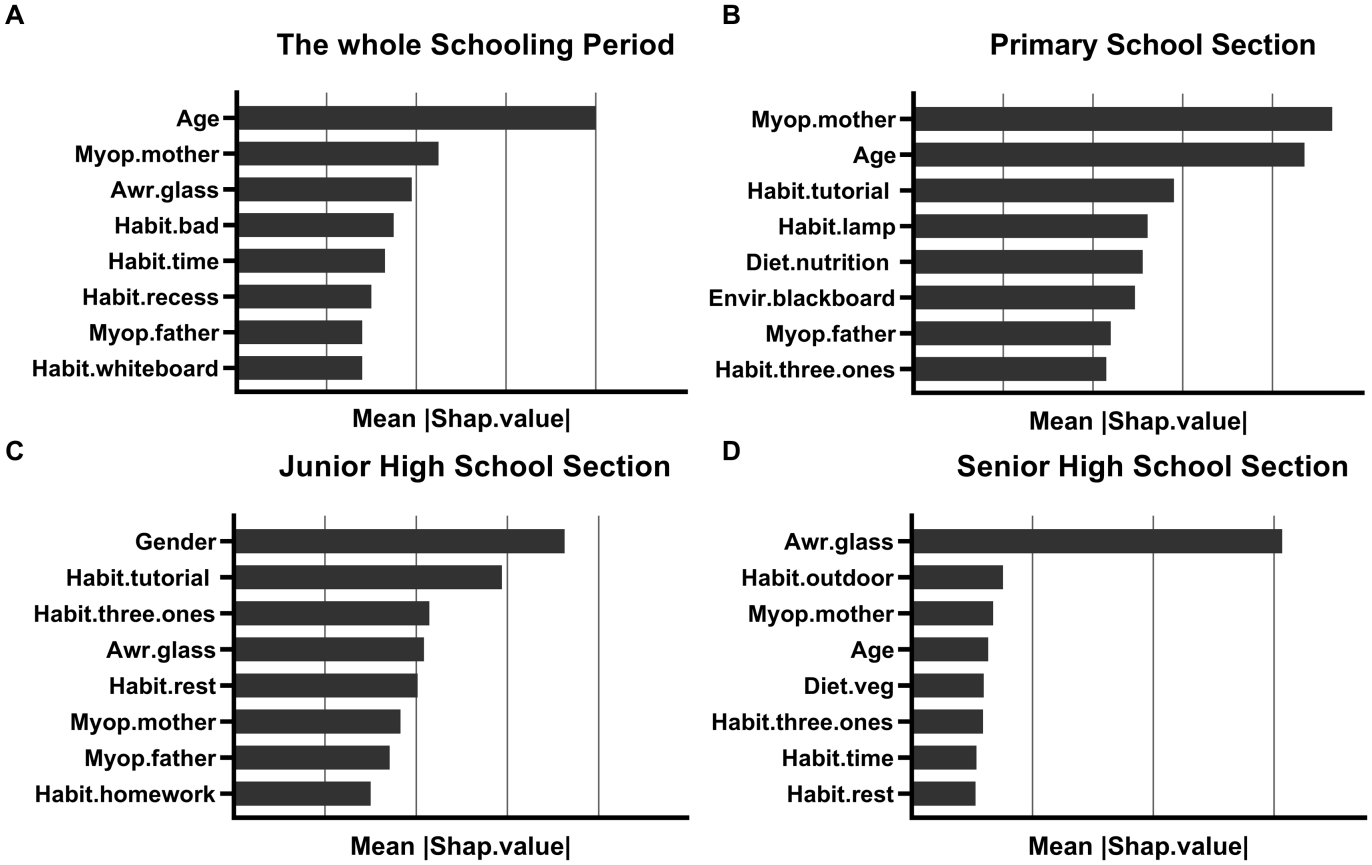
The importance of the characteristics of the model for each school period; **(A)** the whole schooling period; **(B)** the primary school section; **(C)** the junior high school section; **(D)** the senior high school section. The horizontal coordinate represents the average of the absolute values of SHAP values, and the vertical coordinates represent the different characteristics.

While we can determine the specific impact of individual characteristics on the results and the aggregation of individual samples from the SHAP summary plots of the whole schooling period, we cannot determine the relative importance of individual characteristics, which is remedied by the SHAP feature importance plots. The horizontal coordinate of the SHAP feature importance plot is the average of the absolute values of the SHAP values, so we can judge the importance of each feature based on the width of each factor, but we cannot judge whether it is positive or inhibitory (Figure 4).

We build the SHAP feature importance plot by school period to determine the main influencing factors of myopia for each school period. Specifically, the main influencing factors of myopia in students gradually shift from genetic factors to health awareness and behavioral factors as they age. The top three factors influencing the whole schooling period are age, myopic grade of the mother, and Whether myopia requires glasses. In the primary school, most influential factors are the myopic grade of the mother, age, and the extracurricular tutorial subjects weekly. In Junior high school, gender, extracurricular tutorial subjects weekly, and can you do the “three ones” when reading and writing are the most influential factors. During senior high school, the most influential factors are whether myopia requires glasses, daily outdoor activity time, and myopic grade of the mother.

## 4. Discussion

This study combined machine learning algorithms with data from visual acuity screening and questionnaires to construct a classification model for student myopia. Compared to traditional models based on linear regression, machine learning algorithms can effectively avoid the distortions of noisy data in real-world data, while combining variables in a non-linear and highly interactive manner, allowing machine learning algorithms to develop models that are more complex and accurate (27). Each algorithm showed good accuracy on the data set for most school period. Based on the classification model of students’ myopia in each school period, we analyzed the possible influencing factors of students’ myopia in each school section. We found that as the school year progressed, the factors influencing students’ myopia gradually shifted from genetic to behavioral aspects of eye use.

In primary school, the mother’s visual acuity is essential in determining whether a child is myopic. Genetics is probably the most direct explanation for this phenomenon. According to numerous studies, children of myopic parents are more likely to develop myopia (11, 13). However, another possible explanation is that parents share their eye habits and eye environment with their children rather than having a gene for myopia susceptibility (11, 28). Also, since Chinese mothers are more likely than fathers to be in-volved in their children’s lives and help them with their homework, the influence of maternal behavior on children’s myopia is more significant.

While it is surprising that gender is the most critical factor in differentiating myopia in the Junior high school period, considerable research shows gender inequalities in myopia, with females having higher rates of myopia than males (29). This may be because men are more likely than women to participate in outdoor physical activity (30). Puberty may also play an essential role in refractive development, and changes in hormone levels during puberty may influence the onset of myopia (31–34). As junior high school students are just entering puberty, and puberty starts earlier in girls than boys, this may lead to a higher risk of myopia in females.

In the high school, the need to wear glasses for myopia (knowledge of eye health) is the most significant influence on myopia. Studies show that people with more eye health knowledge are at a lower risk of myopia (35, 36). Considering that the onset and progression of myopia is a continuous process that is influenced by factors such as individual eye behavior and the environment in which the eye is used when these influences exceed the threshold required to trigger the onset or progression of myopia, it may result in the onset or progression of myopia. Differences in eye health knowledge reflect, to some extent, differences in individual eye use behavior. For senior students, attention to good eye use habits is an effective measure to prevent myopia and control its development.

It is worth noting that weekly extra-curricular remedial subjects (education) always significantly impact students’ myopia. In China, myopia rates among students continue to rise with the spread of compulsory education and higher education (10). High-pressure education requires children to spend much time doing extra-curricular homework and extra-curricular lessons, which results in more time spent on near-eye behavior and less time spent outdoors. In addition, the proliferation of electronics has meant that students are more likely to stare at screens at weekends than get out of the house. To change this situation, China has tried to reduce myopia rates in recent years through educational reform and by controlling minors’ use of electronic devices. Although the myopia rate among students has been controlled in recent years, it has increased again due to the impact of the epidemic of COVID-19 (37).

In conclusion, genetics, behavior, and the environment when using the eyes, and eye health awareness may all influence myopia in students, and targeted interventions should be implemented according to the physiological development and psychological and behavioral characteristics of students at each school period. External factors that affect myopia vary with age, for primary school students, parents and teachers should actively cultivate good eye habits and provide a good eye environment, as well as the nutrition needed for growth and development. For junior high school students, gender-specific responses are needed. In particular, girls should be encouraged to participate actively in outdoor activities. For high school students, it is important to avoid regular eye use while encouraging them to be active outdoors, provided they have proper eye habits.

There are some limitations to this study. Firstly, there is a possibility of error in the non-cycloplegic refraction. Secondly, the questionnaire is inevitably subject to information bias, such as respondents misunderstanding the questions or recall bias. However, during an epidemic period, vision screening and questionnaire surveys may be the only feasible methods. These erroneous and redundant data may affect the results, but this ‘noise’ can be attenuated by stronger ‘signals,’ as demonstrated in previous studies (22). Finally, the poor performance (Overfitting) of models constructed based on machine learning at the primary school period may be due to the non-inclusion of variables with sufficiently strong correlations, while the limitations of the data itself may also contribute to this.

In conclusion, genetic and environmental factors influence the onset and development of myopia in children and adolescents. Until now, the extent of influence and their relationship is unclear. This study contributes to better precision myopia prevention and control by determining the main influencing factors of myopia in students at each school period through a machine learning approach. However, the results obtained from machine learning models may exhibit biases toward real world, and therefore, professional knowledge and practical considerations should be combined for accurate judgment. Now and in the future, the extensive real-world data generated during the treatment process is a good subject for myopia research, and combined with machine learning algorithms, it can better solve the problem of noisy data and non-linearity, which is essential for us to study the influencing factors of myopia, or predict the development of myopia, and ultimately do myopia prevention and control.

## 5. Conclusion

The results of this study suggest that factors influencing the occurrence of myopia may be different for students in different school periods. Lower-grade students may be more susceptible to genetic or parental behaviors, while eye health awareness and eye use behaviors may influence higher-grade students. However, as a higher dimensional factor, we also find that education always impacts student myopia. Considering those cycloplegic refractive examinations are essential to study the factors that influence myopia, further use of cycloplegia is needed to obtain more accurate data. At the same time, the results of this study are based on the analysis of the data obtained from the questionnaire, and data with a higher degree of confidence are needed to verify this result.

## Data availability statement

The data analyzed in this study is subject to the following licenses/restrictions: this dataset will also be used in a follow-up study by this group and contains private information about the respondent. Requests to access these datasets should be directed to H-JT, zjlxthj666@163.com.

## Author contributions

H-JT and L-LZ: conceptualization. H-JT and Z-MH: methodology, software, and visualization. H-JT: formal analysis and writing—original draft preparation. L-LZ: resources and writing—review and editing. H-JT, BT, Y-MC, and L-LD: data curation. All authors contributed to the article and approved the submitted version.

## Funding

This study was supported by Jiamusi University, special task project for prevention and control of myopia in children and adolescents (JMSUSKZX-ZD003).

## Conflict of interest

The authors declare that the research was conducted in the absence of any commercial or financial relationships that could be construed as a potential conflict of interest.

## Publisher’s note

All claims expressed in this article are solely those of the authors and do not necessarily represent those of their affiliated organizations, or those of the publisher, the editors and the reviewers. Any product that may be evaluated in this article, or claim that may be made by its manufacturer, is not guaranteed or endorsed by the publisher.
